# The role of human papillomavirus in the pathogenesis of head & neck squamous cell carcinoma: an overview

**DOI:** 10.1186/1750-9378-6-4

**Published:** 2011-03-29

**Authors:** Giuseppe Pannone, Angela Santoro, Silvana Papagerakis, Lorenzo Lo Muzio, Gaetano De Rosa, Pantaleo Bufo

**Affiliations:** 1 Department of Surgical Sciences - Section of Anatomic Pathology and Cytopathology - University of Foggia - Foggia - Italy; 2 Department of Surgical Sciences, Section of Anatomic Pathology, 'S. Maria Goretti' Hospital - Latina - Italy; 3 Department of Surgical Sciences - Institute of Anatomic Pathology - University of Bari - Bari - Italy; 4 Department of Otolaryngology - Head and Neck Surgery and Oncology - Medical School, University of Michigan Ann Arbor, Ann Arbor - MI - USA; 5 Department of Surgical Sciences - Section of Oral Pathology - University of Foggia - Foggia - Italy; 6 Section of Pathological Anatomy - Department of biomorphological and functional sciences - University Federico II - Napoli - Italy

## Abstract

Cancer statistics report an increased incidence of OSCC and OPSCC around the world. Though improvements in screening and early diagnosis have dramatically reduced the incidence of this neoplasm in recent years, the 5-year-disease-free survival, is still poor, specially for oropharyngeal cancer, despite the great scientific and financial efforts. Recently, several papers showed that HPV may be involved at least in the pathogenesis of a subgroup of oral and cervical SCC, leading to distinct molecular characteristics compared with HPV-negative ones. Nevertheless, OPSCCs associated with HPV infection seem to show a better prognosis and affect younger patients (< 40 yrs.), especially females. Therefore, there is the need to properly assess oropharyngeal SCC subgroups: 1) not HPV associated/classic oral SCC: less responsive to anticancer drugs: needs novel post-surgical treatment; 2) HPV associated/oral SCC: needs several management options and suitable "target" therapy against the virus, and/or immune-stimulating therapy. Further issues are: 1) the disclosure of putative targets for more efficient molecular therapy, which may work as cervical cancer post-surgical treatment, in anticipation of the effects of "global prevention" performed by WHO anti-HPV vaccination programs; 2) careful identification of precancerous lesions in both sites; dysplasia is currently treated by excisional or ablative procedures, which don't consider the concept of field carcinogenesis. In fact, it is probable that near or far from an excised precancerous lesion new foci of cell transformation may exist, which are not yet macroscopically evident, but, if detected, would put the patient into a high risk subgroup.

Comparing findings reported in the recent literature, the data of this state of the art about HPV might add useful informations concerning oropharyngeal carcinogenesis. Moreover, our review would be useful in order to define novel perspectives of treatment choice for Head & Neck cancer patients, by combining well known chemotherapeutical drugs with new molecular "target" therapy.

## Review

### Epidemiology

HNC is the eighth most common cause of cancer death worldwide. Its incidence varies widely among different regions. In North America and the EU, HNC accounts for 3% to 4% of all cancer diagnoses. Conversely, in Southeast Asia and Africa, HNC accounts for approximately 8% to 10% of all cancers [1]. In EU for 1998 EUCAN reports 42.109 cases of oral and pharynx cancer with 15.744 deaths for men and 11.447 cases with 4.434 deaths for women for a total of 53.556 cases with 20.178 deaths [2]. Up to now OPSCCs represent one of the major health issues, with over 200.000 new cases reported worldwide annually [3]. Although certain subsets of HNC have fallen in parallel with the reduction in smoking, rates of OPSCCs, particularly tongue and tonsillar cancers, have risen steadily by 2.1% and 3.9% among men and women respectively, aged 20-44 years from 1973 to 2001 [4,5]. Though improvements in screening and early diagnosis have dramatically reduced the incidence of these neoplasms in recent years, the 5-year-disease-free survival is still poor, despite the great scientific and financial efforts [6]. The AJCC has accurately defined the primitive anatomic sites of the OSCC: buccal mucosa (2-10%, but in South-East of Asia 40%, for the diffuse habit to chew betel nuts), lip (4-40%, particularly in the lower lip of old patients affected from chronic actinic cheilitis); alveolar ridge (2-18%, including upper and lower gum); retro molar trigonous (2-6%); hard palate (3-6%); floor of the mouth (25%); the ventral two thirds of the tongue (50%, recently raising among young people under 45 years); oropharynx (25%). Squamous cell carcinoma of the oropharynx may originate in the soft palate, tongue base, pharyngeal walls, and tonsils. The tonsils are the most common sub-site followed by the tongue base. In spite of their close proximity tongue base-SCCs, tonsil SCCs and soft palate SCCs have different clinical presentation and treatment outcomes [7]. In particular, tonsil SCCs have significantly better outcome (DSS and DFS) than tongue base SCCs [8].

## Human papillomavirus (HPV) as a risk factor

It is well known that there is a strong association between gene, environment and cancer. Several factors are involved in oral carcinogenesis, such as age, gender, ethnicity, lifestyle, genetic background, status of health and exposure to one or more oncogenic factors [9]. In several epidemiologic studies, tobacco smoking and alcohol consumption have been well documented as major risk factors for oral cancer, with attributable fractions of approximately 90% [10]. However, 15-20% of HNC have no known tobacco or alcohol exposure [11,12].

Thus, other agents, such as viruses, are being investigated. In particular, with regard to viral involvement, it is still highly controversial whether HPV, widely reported as one of the prominent mechanism behind the development of cervical squamous cell carcinoma, can also be considered an aetiological or a malignant risk factor in oral carcinogenesis [13]. According to epidemiological studies, recent improvements in survival with radiotherapy may be due in part to shift in the aetiology of OSCCs [14]. The HPV involvement in oral and oropharyngeal carcinogenesis was first proposed in 1983 by Syrjanen et al. [15] and then supported by several other Authors on the basis of the following evidences: 1) the well-assessed broad epithelial-tropism of HPV; 2) the morphological similarities between oropharyngeal and genital epithelia [16]; 3) the ability of immortalizing human oral keratinocytes *in vitro *[17]; 4) the strongly established etiological role of High Risk HPV in cervical SCC [18,19] and, finally, 5) the detection of HR HPV genotypes in samples of oral squamous cell carcinoma [20].

## HPV Head and Neck Cancer (HPV-HNC): a different entity

In a recent review on epidemiologic and molecular bases, according to Ha and Califano [21], the Authors confirm that HPV plays a role in oral carcinogenesis, and that HPV cancers are specific type of tumours with numerous important differences reported in:- typology of risk patient (generally, never married younger males, < 40 yrs.);

- histological grading (well differentiated cancer) and histotype (possible but no necessary basaloid appearance with a characteristic faster growing);

- response to chemo-radiotherapy, innovative targeted therapy and/or immunostimulating strategies;

- clinical outcome in term of overall survival [22-24]; particularly, patients with HPV-positive HNSCC had a lower risk of dying (meta HR: 0.85, 95% CI: 0.7-1.0), and a lower risk of recurrence (meta HR: 0.62, 95% CI: 0.5-0.8) than HPV-negative HNSCC patients. Site-specific analyses have shown that patients with HPV-positive oropharyngeal tumours had a 28% reduced risk of death (meta HR: 0.72, 95%CI: 0.5-1.0) in comparison to patients with HPV-negative oropharyngeal tumours [25]. Current evidence is strong enough to conclude that HPVs can be transmitted both sexually and non-sexually [26].

Based on recent meta-analysis, besides the classical horizontal transmission during the sexual life, a vertical transmission occurs in approximately 20% of case HPV positive people with HPV-DNA detection in amniotic fluid, foetal membranes, cord blood and placental trophoblastic cells, all suggesting HPV infection in utero, i.e. prenatal transmission [27].

Furthermore, recent studies have proved the existence of a statistically significant synergistic effect between HPV and alcohol. Among heavy alcohol users detected with the virus, the risk of head and neck cancer was statistically significantly increased relative to that of HPV-negative cancer drinkers. Alcohol can biologically modify mucosal tissue, potentially increasing its permeability to viral infection, or it could influence the immune response to HPV [28]. On the other hand the tobacco use, even heavy use, did not play a major role in the synergistic effect between HR HPV and alcohol.

However, the protective mechanism by which HPV infection improves the overall survival and prognosis in oral cancer is not yet clearly understood.

HPVs are a group of host specific DNA virus with a remarkable epithelial cell specificity. More than 120 different HPV genotypes have been identified and almost 45 subtypes, isolated from the low genital tract, have been grouped into high-(HR) and low-risk (LR) HPV types, considering their risk potential to induce an invasive cervical cancer [29].

In a recent study Munoz et al. classify HPV16, 18, 31, 33, 35, 39, 45, 51, 52, 56, 58, 59, 68, 73 and 82 as high risk viruses, detectable in high grade squamous intraepithelial lesions or in invasive cancer; HPV26, 53 and 66 as potential high risk with a not well known oncogenic potential; while types 6, 11, 40, 42, 43, 44, 54, 61, 70, 72, 81 and 89 can be considered as viruses with low oncogenic risk and they can be isolated from low grade epithelial lesions [30].

HPV16, the most common HR HPV type detected in biopsies from women with cervical SCC (55%), was also the most common type detected in biopsies from HNCs (85-95%). In the oropharynx, HPV16 accounted for the overwhelming majority of HPV-positive cases (86.7). Aside from HPV16, other oncogenic HPV types commonly detected in invasive cervical cancer biopsies (e.g., HPV18, 31, 33, 35, 45, 56, 58, and 59) were rarely or never detected in HNC biopsies [31,32].

Conversely, HPV6, which has been designated as ''low-risk'' (LR HPV) or ''non-oncogenic'' to the cervix, was present in a greater number of HNCs than any of the oncogenic types other than HPV16.

It is interesting to note that the classically LR HPVs 6 and 11 subtypes have been found in some tonsillar and laryngeal carcinomas. It is well known that in the rare event of benign laryngeal papillomas undergoing malignant transformation, HPV11 has been most commonly implicated. HPV 6 and 11 have also been implicated in malignancies such as Ackerman's tumour (verrucous carcinoma of the oral cavity). It is clear that in certain rare malignancies, HPV6 and 11 can play a role [33].

Finally, meta-analyses have shown that the HPV subtypes associated with HNC are broadly similar (but not identical) with those seen in cervical carcinoma. This is likely to reflect a difference in life cycles of the different HPV subtypes in different mucosal locations, with an associated difference in mucosal immune responses [34].

## Site by site prevalence of HPV in the Head and Neck region

Up to now, data about HPV prevalence in oral infection, risk factors, genetic pathway and molecular pathogenesis, its potential oncogenic role in oral carcinogenesis are very scanty and still dissenting [35]. Since Syrjanen's observations [15], there have been numerous publications studying HPV DNA detection in OSCCs with rates varying from 0% to 100% of tumours studied [36,37] with higher percentage (also more than the 40% of cases) in those HPV cancers of palatine tonsils and of base of the tongue [38]. This very wide range of HPV prevalence in oral cavity and oropharynx [39-41] and this widespread variability can only be in part due to the cancer site, geographic location and study sample size. Up to now the exact proportion of cancer attributable to viral infection is unclear. The proportion of OSCCs that are potentially HPV-related (cancers of the tongue base and tonsil, including lingual tonsil and Waldeyer's ring) increased in the USA from 1973 to 2004, perhaps as a result of changing sexual behaviours. The IARC Multicenter Study estimated that 18% of oral and oro-pharyngealcancers worldwide are HPV associated [42,43]. There is a general agreement in the current literature as regard the ranking of HPV prevalence according to SCC subsites. HPV infection is most prevalent in OPSCC, followed by LSCCs and, finally, by OSCCs, and not detected in tumors from other HN sites [44,45]. The higher percentage value of viral prevalence, reported and discussed in literature, derives from methodological bias [31], because of the HPV prevalence has been often estimated on different type of squamous cancer arisen from different sites in the head and neck district: oral cavity, Waldeyer area, oro-pharynx, larynx [46]. The footsteps of HPV infection may be traced also in benign oral lesions as condiloma acuminata, common warts, oral papilloma, focal epithelial hyperplasia and in potentially malignant or premalignant erytroplachia, erytro-leukoplakia and leukoplakia, and proliferative verrucous leukoplakia [47]. The exact origin of HPV oral infection is still unclear, contrasting and hypothetical. Because of the close ultrastructural and morphological similarities between oral and vaginal epithelium [16], worldwide scientific research is currently aimed to discover a relationship among HPV, oral and genital malignancies. Postma and Van Heerden have observed a significant association between cervical and oral carcinoma, suggesting that the oral-genital transmitted HPV infection could induce a neoplastic onset, both synchronous and asynchronous, in different mucosal sites [48]. Recent studies have shown that the association between HPV and OSCC is stronger in young males, or in patients characterized by sexual blend or active sexuality or in individuals suffering from genital warts [49].

## Insights into the molecular mechanisms of head and neck HPV carcinogenesis

It is still unknown how persistent infection gives rice to intraepithelial lesions, and, in turn, to invasive HNC. It is known that oncogenic types of HPV may produce non productive-infection and persist in the cells in low number episomic molecules. It is well known that HPVs exert their oncogenic role after DNA integration, gene expression of E5, E6 and E7 loci and p53/pRb host proteins suppression, leading to increased cell proliferation and contributing to carcinogenesis [50,51]. Recently, some Authors suggested that DNA genotyping alone in not sufficient to demonstrate the oncogenic role of HPV in carcinogenesis since integration associated mRNA expression is a necessary step to virus related carcinogenesis. In fact, HPV positive but E6 mRNA negative oropharyngeal cancers showed a prognosis closer to HPV-negative tumors [52].

Although integration of HPV DNA into genomic DNA is a common event in cervical carcinoma and intraepithelial lesions, however, 15-30% of cervical cancer, containing HPV only in the episomal form, shows a plasmidic expression of oncogenic protein E5, E6, E7. The situation in head and neck cancers is not clear, but heterogeneity and the existence of multiple pathways in carcinogenesis is highly likely. Koskinen et al [53] reported that in their series of head and neck HPV16 DNA positive cancers, 48% were integrated, 35% were episomal and 17% were of both mixed episomal and integrated forms. Mellin et al 2002 reported that all cases of HPV+ tonsillar carcinomas harboured HPV-DNA in episomal form [54].

## Molecular detection of HPV

Paradoxically, the low number of HPV DNA copies in integration or episomic status may be underestimated by standard immunopathological studies. When performing the molecular detection of HPV DNA, it is essential that the diagnostic procedures employed are highly sensitive, specific and reliable and it should kept in mind that the efficiency of HPV detection may be affected by several methodological variables. In situ hybridization and in situ oncogenic protein staining techniques have increased sensitivity and specificity of HN diagnostic practices. These techniques have allowed not only detection and identification of low risk/high risk HPV in cytological smears or histopathological immune-sections but, in addition, also the definition of the topographical level of infection, and/or viral integration status (basal layer-integration/upper layers - lytic - episomic phases). Furthermore, these techniques have provided to calculate the copy number per cell (one-two nuclear spot for integration/diffuse nuclear signals for episomic status). In addition, by the routinary IHCthe expression of viral HPV proteins E5, E6, E7 as surrogate markers of HPV infection, and the resulting down-regulation of critical tumor suppressor (p53, pRB, p-16) in histopathologically analyzed HNC can be demonstrated. In any case, we must remember that p16 immunohistochemical positivity is unable to discriminate between HPV integrated vs HPV not integrated OSCCs. IHC and ISH are considered methods with a low sensitivity [46,55], because of the limited availability of antibodies against specific types of HPV (IHC) or the low applicability in clinical routine for the long and hard technical word required (ISH). An highly sensitive broad-spectrum detection of human papillomaviruses should be performed for HPV detection on paraffin-embedded sections collected during diagnostic procedures. Today methods with higher sensitivity (PCR) than the classical immunohistochemical or ISH techniques are able to identify HPV, by detection with type-specific primers or consensus primers [56].

The high sensitive and specific SPF10 HPV DNA test, determined by direct sequencing of PCR fragments and genotyping assay, as good screening test, can be performed for HPV detection on paraffin-embedded sections collected during diagnostic procedures as good screening test [57].

PGMY/GP nested PCR system is able to perform consistently at a high level of sensitivity, namely 0.1-1 copy per cell input [58].

However the possibility of overestimation or underestimation of HPV positivity due to technical limitations, the absence of standardization in collection, storing and analyses of tissue samples, the impact in the clinical practice, the cost and the commercial availability are important aspects to consider, in order to establish the exact role of HPV in oral and oropharyngeal lesions and its real tumoral frequency in an anatomical region where HPV has a yet high prevalence.

In clinical setting, nowadays we have to perform a sensible, specific and accurate HPV test. In case of false negative results, patients are potentially deprived of important curative tools i.e. radio and chemotherapy. Similarly, false positive patients are potentially deprived of another curative tool: the surgery. Only standardized technical procedures could assist clinicians to provide effective diagnostic test, innovative treatment and more efficient screening systems for OSCC patients. A specific panel of different HPV types, possibly including LR and HR-viruses, should be defined in order to prepare adequate vaccine reducing the risk of HPV-positive oral and oropharyngeal cancers in male and female population and in order to detect an infective pathogenesis in oral and oropharyngeal neoplastic samples.

## The role of p16^INK4a ^alterations, p-53 mutations and molecular markers of HPV infections in HN-SCC

### p16INK4a and viral load

The INK4A locus, harboring the p16INK4A gene, is a major aberration hotspot in oral and oropharyngeal carcinoma. The p16INK4A gene functions as negative regulator of the cell cycle progression through its inhibition of cdk4/6 and subsequent blockage of the cyclin-dependent phosphorylation of the Rb [59]. Genetic alterations of p16INK4A lead to loss of control of the restriction point in the G1 phase of the cell cycle and favour cellular transformation [60]. It is also found that p16INK4A expression loss defines a subgroup of oral cancer patients with worse clinical outcome [61].

The current scientific research is aimed at deciphering the molecular alterations involved in p16INK4A down-regulation and to assess the prognostic implications of p16INK4A gene alterations in HNC in order to establish its impact on staging and prognosis compared with the conventional clinical staging parameters [62].

The instability of the CDKN2a/INK4 locus located on 9p21 is reported to be high in carcinoma and the major inactivation of the p16INK4A/p14ARF genes results from promoter methylation, homozygous deletion, loss of heterozygosity and intragenic mutation [63].

These genetic and epigenetic alterations have been detected frequently in a variety of human cancers, including head and neck cancer.

According to recent studies, HPV-positive HNCs have intact p16 gene and wild type p53 compared to HPV negative ones, harbouring frequent p16 and p53 gene alterations [64]. This is the reason of their chemo-radio-sensitivity, since apoptotic pathway are preserved. Anyway HPV infections have been also demonstrated in the p53 mutated group of HN cancer, suggesting the concept that an HPV may super-infect an already mutated cancer cell [65].

For many years, the main prognostic factors of SCC have been the conventional grading, staging and site of tumor. According to recent studies, the viral load of HPV in HNCs as well as p16 expression could be defined the most relevant prognostic markers in HPV positive OSCCs, surpassing the significance of the classical histopathological parameters, and it should be considered for inclusion into official staging system of HNSCC. The viral load of HPV in HNCs appears to vary considerably as reported in a recent update[66].

Available data suggest that oral and oropharybgeal cavity HPV viral load measured by DNA is lower than in the cervix. Tonsillar cancer appears to show a wide variation in HPV copy number. The study from Mellin et al 2002 [54] detected 10 - 15,400 (median of 190) HPV 16 copies per beta-actin copy from eleven HPV 16+ tumours. The data suggested that a higher viral load could be a favourable prognostic indicator and that tumours with episomal DNA had larger tumours than patients with mixed or integrated forms of viral DNA. This shows that the higher copy number of episomal viral DNA was able to induce more rapid growth, perhaps by higher expression of the viral oncogenes. Weinberger et al [67] demonstrated that HPV 16 viral load measured by real time PCR, detection of HPV infection by ISH and p16 expression valued by immunohistochemistry could be used as a *gold standard *test to classify head and neck cancers into 3 distinct profiles: Class I, HPVnegative, p16 low, 0.0 copies; Class II, HPV+, p16 low, 3.6 copies; and Class III, HPV+, p16 high, 46 copies; Class IV, HPV-negative; p16 + Of note, was that Class III tumours had a significantly increased 5 year survival, increased disease-free survival rate and decreased local recurrence rate, compared to tumours in the other classes (Table 1).

**Table 1:** Weinberger classification (2006)

Weinberger classification (2006)*	
**HPV status; p16 expression**	**Median HPV DNA viral load (copies HPV 16/human genome)**

Class I, HPV-; p16 low	0.0 copies - Class I

Class II, HPV+; p16 low	3.6 copies - Class II

Class III, HPV+; p16 high	46.0 copies - Class III

Class IV, HPV-; p16 +	

For further detail see the text

*according to the contents reported in: Weinberger PM, et al.: Molecular classification identifies a subset of human papillomavirus--associated oropharyngeal cancers with favorable prognosis. J Clin Oncol 2006, 24(5):736-47 [67]

Nowadays, the goal of the scientific research is to find new biological markers able to identify set(s) of genes involved in oro-pharyngeal carcinogenesis. This is necessary to evaluate the "tumor fingerprint" that defines the biological behavior of the each single neoplasm. In the last decade the scientific research has shown a growing interest on several biological markers, expressed by cancerous cells in order to use them as indexes of OSCC progression and aggressiveness, also in relation to HPV-DNA detection. The investigation of oncogenic gene expression in HPV-related OSCC and the study of its potential value as predictor of neoplastic progression and clinical outcome could allow to characterize a possible evolutive morphologycal profile of oral cancer and its precancerous lesions. As a rule, the expression of HPV markers and surrogate markers of HPV infection can be easily evaluated by IHC. Different antigens should be used including virus related (capsidic antigens, E5-E6-E7 proteins), and virus induced and/or altered host proteins (p16^INK4a^, pRb, Cyclin proteins, p-53).

**Figure 1:**
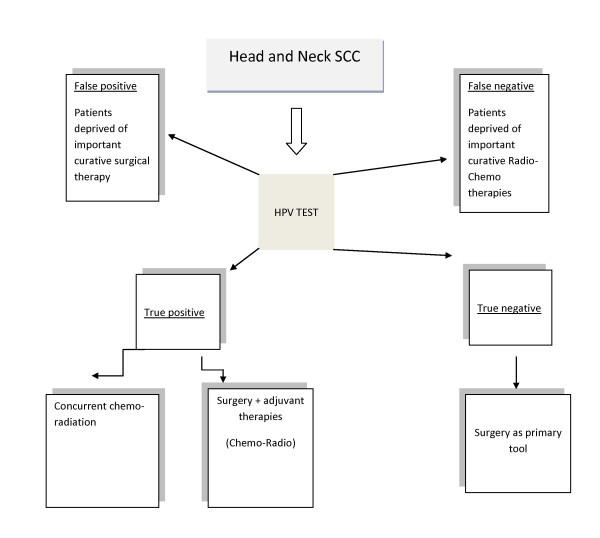
**Flow chart**. Dramatic consequences on the Head & Neck cancer therapeutic management of missing or misinterpreting HPV test are described.

Several authors have emphasized that a hallmark of the presence of HPV in cancer could be found in p16 nuclear or cytoplasmic overexpression, so that p16 could be considered a useful surrogate marker for HPV [66,68]. Recent reports have proved that HPV+ OSCCs harbour frequently unmethylated CDKN2A promoter as compared to smoke related OSCC frequently methylated [69]. The employment of biomarkers improve the current diagnostic tools but also can contribute indirectly to therapeutics as predictor of choice for the correct clinical management. Therefore, there is the need to properly assess OSCC subgroups: 1) not HPV associated/classic OSCCs that are less responsive to anticancer drugs and need novel post-surgical treatment; 2) HPV associated OSCCs that have a lower risk of dying and recurrence and need several management options and suitable "target" therapy and/or immune-stimulating strategies. Currently, the group of HPV-negative OSCC patients have to be surgically treated, since the intrinsic chemo-resistance of their tumors; the group of HPV+ OSCC patients should be treated by surgery plus adjuvant radio-chemotherapy, or alternatively by concurrent radio-chemoterapy and by salvage surgery only for patients non-responders to induction radiochemotherapy (Figure 1).

### Other markers in differential diagnosis between HPV-positive and HPV-negative cancer

On the basis of these tendencies, some Authors [70,71] have identified specific biomarkers, by using c-DNA microarrays, in order to distinguish HPV+ versus HPV- OSCCs, to improve the current diagnostic tools but also to contribute indirectly to therapeutics as predictor of choice for the correct clinical management (Table 2). In a recent microarray based study of Pyeon D et al [70] two main subgroups of head-neck cancer (HNC) have been identified: HPV+HNC and HPV- HNC. Not surprisingly, closes classes were the two HPV+ cancers, HPV+ HNC and HPV+ cervical cancer. The substantial correlation between HPV+ HNCs and HPV+ cervical cancers implies a substantial role for virus-dependent, tissue-independent factors in gene expression changes. The study described genome wide expression profiling of HNCs, cervical cancers, and site-matched normal epithelial samples. More specifically, in tumor/normal comparisons HPV+ HNC, HPV- HNC, and cervical cancer all were up-regulated relative to normal controls for a gene set I, including keratins (*KRT8, KRT17, KRT18*), caveolin (*CAV2*), IFNα-inducible protein 6-16 (*G1P3*), matrix metallopeptidase 12 (*MMP12*), collagens (*COL4A1, COL4A2*), and phospholipid scramblase 1 (*PLSCR1*), and down-regulated for another set II, including other keratins (*KRT4, KRT13, KRT15*), programmed cell death 4 (*PDCD4*), protein tyrosine kinase 6 (*PTK6*), epithelial membrane protein 1 (*EMP1*), extracellular matrix protein 1 (*ECM1*), interleukin 1 receptor (*IL1R2*), and transglutaminase 3 (*TGM3*). Relative to HPV-HNC HPV+HNC and cervical cancer showed significantly increased expression of gene set III, including PC4/SFRS1-interacting protein 1 (*PSIP1*), V-myb (*MYB*), synaptogyrin 3 (*SYNGR3*), SWI/SNF-related, matrix-associated, actin-dependent regulator of chromatin (*SMARCA2*), synaptonemal complex protein 2 (*SYCP2*), p16 (*CDKN2A*), lymphoid-specific heli-case (*HELLS*), and testicular cell adhesion molecule 1 (*TCAM1*), whereas expression was decreased for gene set IV, including parathyroid hormone-like hormone (*PTHLH*), cortactin (*CTTN*), kallikreins (*KLK8, KLK10*), cyclin D1 (*CCND1*), caveolin 1 (*CAV1*), and defensin β4 (*DEFB4*). E7 oncoproteins of high-risk HPVs induce DNA replication and mitosis by multiple mechanisms, including interacting with pRb, HDACs, and other factors to activate cell cycle-regulated transcription factors such as E2F [72]. A significant subset of cell cycle-regulated genes was differentially expressed in HPV+ HNC and cervical cancer relative to HPV-HNC. HPV-HNCs up-regulated, relative to HPV+ cancers, a small set of cell cycle-specific genes, including cyclin D1/D2 (*CCND1 *and *CCND2*; G1 associated) and cyclin A1 *(CCNA1*). By contrast, HPV+ cancers up-regulated, relative to HPV-HNC, a much larger set of cell cycle-specific genes such as cyclin E2 (*CCNE2*; G1 associated), cyclin B1 (*CCNB1*; G2 associated), and multiple MCMs. Among these, many genes that enhance DNA replication and cell mitosis, including proliferating cell nuclear antigen (*PCNA*), *E2Fs, cdc2, cdc7*, and *MCMs *were significantly up-regulated in HPV+ HNC and cervical cancer relative to HPV- HNC, implying that the HPV+ cancers were more active in cell division. The most striking difference in cell cycle regulatory gene expression was seen with *cyclin D1/CCND1 *and *p16/Ink4a/CDKN2A*. In HPV+ cancers, p16 was expressed at high levels and cyclin D1 at low levels, with the converse in HPV- cancers. A recent immunohistochemical study examining just six cell cycle proteins in HNCs confirmed that these changes in p16 and cyclin D1 expression correlate with HPV status and extend to the protein level [73]. In HPV+ cancers, p16 up-regulation and cyclin D1 down-regulation are thought to be a consequence of feedback loops from E7 inhibition of Rb activity. For many HPV- cancers, including HPV- HNCs, reduced p16 expression correlates with p16 promoter hypermethylation, whereas cyclin D1 overexpression is linked to gene amplification. Such a virus-induced, highly proliferative state may be responsible for the greater responsiveness of HPV+ HNCs to radiation therapy [74]. Recently [70], up-regulation of novel testis antigens in HPV+ cancers has been showed. Genes highly up-regulated in HPV+ cancers relative to HPV- HNC included two testis specific genes not normally expressed in somatic cells, *SYCP2 *and *TCAM1*. Cell line studies showed that SYCP2 and TCAM1 expression are synergistically up-regulated by E6 and E7. The Peyon's study [70] found that HPV+ cancers over-express novel testis antigens *SYCP2, STAG3*, and *TCAM. STAG3 *and *SYCP2*, an *SYCP1 *homologue, are components of the meiotic synaptonemal complex that promotes recombination, and *SYCP1 *expression induces formation of a synaptonemal complex-like structure. Aberrant expression of these meiosis-specific proteins in HPV+ cancers may contribute to the genomic instability induced by high-risk HPVs and to further genetic changes during HPV-associated cancer development. According to Slebos' model [71], important biological markers expressed by tumoral cells (e.g. MIB-1, PCNA, cyclin D1, p18 e CDC7, transcription factors, as TAF7L, RFC4, RPA2, and TFDP2, adhesion molecules as TCAM1) could be used as indexes of oral-cervical SCC progression and aggressiveness, also in relation to HPV-DNA detection. Soares et al. found an high expression of PCNA in oral and cervical SCC HPV-positive, reconducted to HPV-related oncogenetic promotion [75]. Other studies have been shown an aberrant expression of cyclin D1 in the majority of oral and cervical SCC: this situation is resulted tightly related to their un-favourable prognosis [76]. Immunoistochemical detection of MIB1 (Ki-67) has been suggested in order to determine proliferation index in oral and cervical SCC [77].

**Table 2:** Cell cycle related genes

Cell cycle related genes	
**HPV positive cancers**	**HPV negative cancers**
**• P-16**	• Cyclin D1/D2
**• P-18**	• Cyclin A1
**• Cyclin B1**	
**E2F family:**	
**• MCMs**	
**• Cyclin E**	
**• PCNA**	

For further detail see the text

HPV+ HNCs show a wide range of cell cycle related proteins, p-16, p-18, Cyclin B1, and the E2F family (MCMs, Cyclin E, PCNA) as compared to HPV-negative HNCs, showing a more restricted panel of cell cycle proteins, Cyclin D1/D2, Cyclin A1. According to the studies from Heidelberg University group, most HPV++ designated OPSCCs (cancers with high viral load of HPV16 mRNA) showed reduced pRb, low cyclin-D1 and p-53 and up-regulated p16^INK4a^; in contrast, HPV+ designated tumors (cancers with low viral load of HPV16 mRNA showed normal pRb, increased Cyclin D1 and p-53, and reduced p16^INK4a^, a pattern typical for HPV-negative tumors; LSCCs showed only the pattern for HPV negative tumors [78]. All these findings might add useful informations concerning oropharyngeal and cervical carcinogenesis and, finally, offer a novel perspective of treatment choice for the patients, by combining well known chemotherapeutical drugs with new molecular "target" therapy.

## HPV detection as evaluated by cytopathological and molecular methods. Screening program and the potential role of saliva

The association of head and neck cancers with clinically significant morbidity and disfiguration makes early detection of the diseases and biomarkers to identify individuals at high risk of great importance. However, most of these markers have been identified either in cancer cell lines or in biopsy specimens from late invasive and metastatic cancers. Moreover, the invasive nature of a biopsy makes it unsuitable for cancer screening in high-risk populations. Exfoliative cytology may be a less invasive method for oral cancer detection. In some recent scientific research has been assessed that HR HPV types detected in oral exfoliated cells could be used as a predictive biomarker of oropharyngeal cancer risk associated with HR HPV infection [28,79], because an association between the detection of HR HPV types in oral exfoliated cells and the presence of HR HPV types in tumor tissue has been established. HR HPV DNA detected in the oral exfoliated cell samples may originate from HPV-positive tumor cells, from any associated HR-HPV infection that led to the development of oro-pharyngeal cancer, or from an independent HR HPV infection. Detection of HR HPV DNA may help identify individuals, including those with 1) any genetic predisposition to acquire HR HPV infection and/or 2) a limited immunologic ability to eliminate the virus, who are at risk for the development of oropharyngeal cancer because they are susceptible to HR HPV infection in the head and neck area. Whether oral exfoliated HR HPV status is predictive of cancer before invasion or progression in patients with HNC is unknown, but the answer could be clarified easily and inexpensively by repeated assessment in HR HPV cases over time. The use of oral exfoliated cells could be extended to evaluate other molecular markers of early carcinogenesis in head and neck tumors in addition to HPV, including alterations in tumor suppressor gene pathways, changes in gene expression profiles, and microsatellite markers to increase their power to predict early-stage cancer. Serologic assessment of HPV in HNC may be less sensitive than assessment of oral exfoliated cells. A blood draw is less acceptable to patients, more difficult to administer and preserve, and currently does not allow the evaluation of a wide variety of HPV types. Therefore, HPV seropositivity is potentially indicative not only of current oral infection but also of any past infection not limited to the oral cavity or oropharynx, including infections in the anogenital area that are thought to be the most likely source of HPV in the majority of individuals with an HPV-positive serologic test. Finally, the detection of HR HPV in oral exfoliated cells, its plasmid versus integrated state, as well as specific integration sites and gene expression patterns may serve as clonal markers to monitor the presence of residual tumor after surgery or radiation, cancer recurrence, and progression. The availability of an easily performed cytological test is essential for disease prevention, early diagnosis and tumor re-staging but we have to remember that exfoliated cancer cells tend to correlate with tumor burden and with lower rates of detection seen in those with minimal or early disease. This suggests an imperative need for developing new diagnostic tools that would improve early detection. The identification of molecular markers in body fluids that would predict the development of cancer in its earliest stage or in precancerous stage would constitute such a tool. Whole human saliva is easily collected in the clinic in a non-invasive, on-demand manner and in relatively large, easily stored quantities, making it an optimal bodily fluid for clinical diagnostics. Diagnosis of oral cancer could benefit greatly from the development of whole saliva-based clinical tests given the physical proximity of the site of cancer development with the diagnostic fluid.

Moreover we have to remember that saliva can contain different types of HPV reservoirs, and HPV DNA may originate from the following sites:

- HPV-positive tumor cells

- HR HPV infection that led to the development of oropharyngeal cancer

- from an independent HR HPV infection;

- phlogistic cells, as giant cell or lymphocytes;

- oral epithelium surrounding tumor: normal or dysplastic mucosa;

- tonsillar crypts.

Generally, the sensitivity of an HPV test in saliva has been reported as low, whereas the specificity is very high. Recent studies report that HPV16 was detected in 50% of the saliva rinse samples from HNSCC patients with detectable HPV16 level in their tissues, in 18% of saliva rinse samples from patients with HPV16 negative primary HNC and in 2.8% of the normal controls. Using a cutoff of HPV 16 >0.001 copies/cell in saliva rinse, the test yielded a sensitivity of 30.4% and a specificity of 98.3%. HPV16 DNA in saliva rinses can reflect HPV16 status of primary HNSCC. As a screening method quantitative analysis of HPV16 DNA in salivary rinses allows for detection of HPV-related HNC; however, specific limitations exist that prevent the application of this as a screening technique for a broad population [80].

Quantitative measurement of salivary HPV16 DNA can be a promise for surveillance and early detection of recurrence. In fact, a very interesting paper proved that the presence of HPV DNA in convalescent salivary rinses is an adverse prognostic marker in HN squamous cell carcinoma, because HPV16 presence in follow-up salivary rinses preceded clinical detection of disease recurrence by an average of 3.5 months [79]. Recent discovery by microarray technology that a large panel of human mRNA exists in saliva suggests a novel clinical approach, salivary transcriptome diagnostics, for applications in disease diagnostics as well as for normal health surveillance [81,82]. It is a high-throughput, robust, and reproducible approach to harness RNA signatures from saliva. Understanding the profile of molecular changes in any particular HPV+/HPV- cancer will be extremely useful because it will become possible to correlate the resulting phenotype of that cancer with molecular events. One of the scientific goals is to construct risk models to facilitate assigning the appropriate salivary transcriptome-based diagnosis for patients' specific cancer risk.

Synthetically, saliva allows to:

- detect HR HPV in oral cells

- identify its plasmidic vs integrated state

- define gene expression patterns by:

Genomic Microarrays

EpiGenomic Microarrays

Proteomic analysis

- be easily used in general population study

- be easily used also in selected high rish populations (immune-depression/immune-suppression)

- help in decision making as regard the choice of therapy

- monitor presence of residual tumor after therapy (cancer progression)

## Conclusions: 'the need of a global immunization programme'

Recent reviews were performed in order to establish the evidence for HPV-related malignant disease in regions other than the cervix. One of this studies found sufficient evidence to support a global immunization programme against HPV, irrespective of gender and geography, to help to achieve a reduction in HPV-related malignant diseases in the future [83].

## List of Abbreviations

OPSCC: Oro-Pharyngeal Squamous Cell Cancers; OSCC: Oral Squamous Cell Cancer; HPV: Human Papillomavirus; SCC: Squamous Cell Carcinoma; WHO: World Health Organization; HNC: Head and Neck Cancer; EU: European Union; AJCC: American Joint Comittee on Cancer; DSS: Disease Specific Survival; DFS: Disease Free Survival; HR HPV: High Risk HPV; LR HPV: Low Risk HPV; IARC: International Agency on Treatment of Cancer; LSCC: Larynx Squamous Cell Cancer; HN: Head and Neck; IHC: Immunohistochemistry; ISH: in situ hybridization; PCR: Polymerase Chain Reaction.

## Competing interests

The authors declare that they have no competing interests.

## Authors' contributions

GP conceived of the study; PB, AS and SP have made substantial contribution to its design; AS and GP have participated in the acquisition of literature data; GP together with AS and PB helped in the coordination of this work; AS has been involved in drafting the manuscript and in its sequences'alignment, too; finally, PB, GDR and LLM have revised it critically for important intellectual content and they have given final approval of the version to be published. All Authors read and approved the final manuscript.

## References

[B1] Santarelli A Lo Russo L Bambini F Campisi G Lo Muzio L New perspectives in medical approach to therapy of head and neck squamous cell carcinoma Minerva Stomatologica 2009 58 9 445 52 19893469

[B2] Landis SH Murray T Bolden S Wingo PA Cancer statistics CA Cancer J Clin 1998 48 6 29 10.3322/canjclin.48.1.6 9449931

[B3] Horner MJ R L Krapcho M Neyman N Aminou R Howlader N Altekruse SF Feuer EJ Huang L Mariotto A Miller BA Lewis DR Eisner MP Stinchcomb DG Edwards BK eds SEER Cancer Statistics Review, 1975-2006 1975 National Cancer Institute http://seer.cancer.gov/csr/1975_2006/Bethesda, MD based on November 2008 SEER data submission, posted to the SEER web site, 2009

[B4] Frisch M Changing patterns of tonsillar squamous cell carcinoma in the United States Cancer Causes Control 2000 11 6 489 95 10.1023/A:1008918223334 10880031

[B5] Shiboski CH Schmidt BL Jordan RC Tongue and tonsil carcinoma: increasing trends in the U.S. population ages 20-44 years Cancer 2005 103 9 1843 9 10.1002/cncr.20998 15772957

[B6] World Health Organization The World Oral Health Report 2003 2003 Geneva: World Health Organization 6 7

[B7] Pathak KA Al Hajjaj H Viallet NV Sutherland DS Kerr PD Nason RW Squamous cell carcinoma of the oropharynx: influence of site of primary tumor Oral abstracts/Oral Oncology 2009 Supplement 3 (1) 73 10.1016/j.oos.2009.06.136

[B8] Jaber JJ Moreira J Canar WJ Bier-Laning CM A 25-Year Analysis of Veterans Treated for Tonsillar Squamous Cell Carcinoma Arch Otolaryngol Head Neck Surg 2009 135 11 1147 115 10.1001/archoto.2009.164 19917929

[B9] Llewellyn CD Johnson NW Warnakulasuriya KA Risk factors for oral cancer in newly diagnosed patients aged 45 years and younger: a case-control study in southern England J Oral Pathol Med 2004 33 525 32 10.1111/j.1600-0714.2004.00222.x 15357672

[B10] Castellsague X Quintana MJ Martinez MC Nieto A Sanchez MJ Juan A The role of type of tobacco and type of alcoholic beverage in oral carcinogenesis Int J Cancer 2004 108 741 9 10.1002/ijc.11627 14696101

[B11] Gillison ML Shah KV Human papillomavirus-associated head and neck squamous cell carcinoma: mounting evidence for an etiologic role for human papillomavirus in a subset of head and neck cancers Curr Opin Oncol 2001 13 183 8 10.1097/00001622-200105000-00009 11307062

[B12] Jo S Juhasz A Zhang K Ruel C Loera S Wilczynski SP Human Papillomavirus Infection as a Prognostic Factor in Oropharyngeal Squamous Cell Carcinomas Treated in a Prospective Phase II Clinical Trial Anticancer Res 2009 29 5 1467 74 19443352 PMC3582681

[B13] Chaudhary AK Singh M Sundaram S Mehrotra R Role of human papillomavirus and its detection in potentially malignant and malignant head and neck lesions: updated review Head Neck Oncol 2009 1 1 22 10.1186/1758-3284-1-22 19555477 PMC2706235

[B14] Chaturvedi AK Engels EA Anderson WF Gillison ML Incidence trends for human papillomavirus-related and -unrelated oral squamous cell carcinomas in the United States J Clin Oncol 2008 26 612 619 10.1200/JCO.2007.14.1713 18235120

[B15] Syrjänen KJ Pyrhönen S Syrjänen SM Lamberg MA Immunohistochemical demonstration of human papilloma virus (HPV) antigens in oral squamous cell lesions Brit J Oral Surg 1983 21 2 147 53 6307342 10.1016/0007-117x(83)90060-4

[B16] Thompson IO van der Bijl P van Wyk CW van Eyk AD A comparative light-microscopic, electron-microscopic and chemical study of human vaginal and buccal epithelium Arch Oral Biol 2001 46 1091 1098 10.1016/S0003-9969(01)00082-6 11684027

[B17] Shin KH Min BM Cherrick HM Park NH Combined effects of human papillomavirus-18 and N-methyl-N'-nitro-N-nitrosoguanidine on the transformation of normal human oral keratinocytes Mol Carcinog 1994 9 76 86 10.1002/mc.2940090205 8142012

[B18] zur Hausen H de Villiers EM Gissmann L Papillomavirus infections and human genital cancer Gynecol Oncol 1981 12 S124 128 10.1016/0090-8258(81)90067-6 6273261

[B19] Bosch FX Lorincz A Muñoz N Meijer CJ Shah KV The causal relation between human papillomavirus and cervical cancer J Clin Pathol 2002 55 244 265 11919208 10.1136/jcp.55.4.244 PMC1769629

[B20] Miller CS Johnstone BM Human papillomavirus as a risk factor for oral squamous cell carcinoma: A meta-analysis, 1982-1997 Oral Surgery, Oral Medicine, Oral Pathology, Oral Radiology and Endodontology 2001 91 6 622 635 10.1067/moe.2001.115392 11402272

[B21] Ha PK Califano JA The role of human papillomavirus in oral carcinogenesis Crit Rev Oral Biol Med 2004 15 188 96 10.1177/154411130401500402 15284184

[B22] Li W Thompson CH O'Brien CJ McNeil EB Scolyer RA Cossart YE Human papillomavirus positivity predicts favourable outcome for squamous carcinoma of the tonsil Int J Cancer 2003 106 553 8 10.1002/ijc.11261 12845651

[B23] Ishikawa H Mitsuhashi N Sakurai H Maebayashi K Niibe H The effects of p53 status and human papillomavirus infection on the clinical outcome of patients with Stage IIIB cervical carcinoma treated with radiation therapy alone Cancer 2001 91 80 9 10.1002/1097-0142(20010101)91:1<80::AID-CNCR11>3.0.CO;2-E 11148563

[B24] Mellin H Friesland S Lewensohn R Dalianis T Munck-Wikland E Human papillomavirus (HPV) DNA in tonsillar cancer: clinical correlates, risk of relapse, and survival Int J Cancer 2000 89 300 4 10.1002/1097-0215(20000520)89:3<300::AID-IJC14>3.0.CO;2-G 10861508

[B25] Fakhry C Westra WH Li S Improved survival of patients with human papillomavirus-positive head and neck squamous cell carcinoma in a prospective clinical trial J Natl Cancer Inst 2008 100 4 261 9 10.1093/jnci/djn011 18270337

[B26] D'Souza G Kreimer AR Case-control study of human papillomavirus and oropharyngeal cancer N Engl J Med 2007 356 19 1944 56 17494927 10.1056/NEJMoa065497

[B27] Syrjänen S Current concepts on human papillomavirus infections in children APMIS 2010 118 6-7 494 509 20553530 10.1111/j.1600-0463.2010.02620.x

[B28] Smith EM Ritchie JM Summersgill KF Hoffman HT Wang DH Haugen TH Turek LP Human Papillomavirus in Oral Exfoliated Cells and Risk of Head and Neck Cancer Journal of the National Cancer Institute 2004 96 6 15026470 10.1093/jnci/djh074

[B29] Campisi G Giovannelli L Controversies surrounding human papillomavirus infection, head & neck vs oral cancer, implication for prophylaxis and treatment Head and Neck Oncology 2009 I 8 Commentary 10.1186/1758-3284-1-8 PMC2673223 19331691

[B30] Muñoz N Bosch FX de Sanjosé S Herrero R Castellsagué X Shah KV International Agency for Research on Cancer Multicenter Cervical Cancer Study Group Epidemiologic classification of human papillomavirus types associated with cervical cancer N Engl J Med 2003 348 6 518 27 12571259 10.1056/NEJMoa021641

[B31] Kreimer AR Human papillomavirus types in head and neck squamous cell carcinomas worldwide: a systematic review Cancer Epidemiol Biomarkers Prev 2005 14 2 467 75 10.1158/1055-9965.EPI-04-0551 15734974

[B32] Termine N HPV in oral squamous cell carcinoma vs head and neck squamous cell carcinoma biopsies: a meta-analysis (1988-2007) Ann Oncol 2008 19 10 1681 90 10.1093/annonc/mdn372 18558666

[B33] Ostwald C Rutsatz K Schweder J Schmidt W Gundlach K Barten M Human papillomavirus 6/11, 16 and 18 in oral carcinomas and benign oral lesions. Human papillomavirus infections and oral tumors Medical Microbiology and Immunology 2002 192 3 145 148 10.1007/s00430-002-0161-y 12920590

[B34] Stina Syrjänen Human papillomavirus infections and oral tumors Medical Microbiology and Immunology 2003 192 3 123 128 10.1007/s00430-002-0173-7 12920585

[B35] Gillison ML Koch WM Capone RB Spafford M Westra W Wu L Evidence for a Causal Association Between Human Papillomavirus and a Subset of Head and Neck Cancers J Natl Cancer Inst 2000 92 9 709 720 10.1093/jnci/92.9.709 10793107

[B36] Mork J Human papillomavirus infection as a risk factor for squamous-cell carcinoma of the head and neck N Engl J Med 2001 344 15 1125 31 10.1056/NEJM200104123441503 11297703

[B37] Hansson BG Strong association between infection with human papillomavirus and oral and oropharyngeal squamous cell carcinoma: a population-based case-control study in southern Sweden Acta Otolaryngol 2005 125 12 1337 44 10.1080/00016480510043945 16303684

[B38] Syrjanen S HPV infections and tonsillar carcinoma J Clin Pathol 2004 57 5 449 55 10.1136/jcp.2003.008656 15113849 PMC1770289

[B39] Hennessey PT Westra WH Califano JA Human papillomavirus and head and neck squamous cell carcinoma: recent evidence and clinical implications J Dent Res 2009 88 4 300 6 10.1177/0022034509333371 19407148 PMC3317947

[B40] Tachezy R Klozar J Rubenstein L Smith E Saláková M Smahelová J Demographic and risk factors in patients with head and neck tumors J Med Virol 2009 81 5 878 87 10.1002/jmv.21470 19319944

[B41] Klussmann JP Weissenborn SJ Wieland U Dries V Kolligs J Jungehuelsing M Prevalence, distribution, and viral load of human papillomavirus 16 DNA in tonsillar carcinomas Cancer 2001 92 11 2875 84 10.1002/1097-0142(20011201)92:11<2875::AID-CNCR10130>3.0.CO;2-7 11753961

[B42] Petersen PE Oral cancer prevention and control--the approach of the World Health Organization Oral Oncol 2009 45 4-5 454 60 10.1016/j.oraloncology.2008.05.023 18804412

[B43] Herrero R Castellsague X Pawlita M Human papillomavirus and oral cancer: the international agency for research on cancer multicenter study J Natl Cancer Inst 2003 95 23 1772 1783 14652239 10.1093/jnci/djg107

[B44] Machado J Reis PP Zhang T Simpson C Xu W Perez-Ordonez B HPV detection in head and neck cancer using the roche linear array HPV genotyping test Oral abstracts/Oral Oncology 2009 Supplement 3 (1)

[B45] Pannone G Bufo P Santoro A Papagerakis SM Rubini C Lo Muzio L Double demonstration of oncogenic HPV DNA and HPV-E7 protein in 8.57% of oral cancers. Preliminary report Oral abstracts/Oral Oncology 2009 Supplement 3 (1)

[B46] Scapoli L Palmieri A Rubini C Martinelli M Spinelli G Ionna F Low prevalence of human papillomavirus in squamous-cell carcinoma limited to oral cavity proper Mod Pathol 2009 22 3 366 72 10.1038/modpathol.2008.180 18978731

[B47] Szarka K Tar I Fehér E Gáll T Kis A Tóth ED Progressive increase of human papillomavirus carriage rates in potentially malignant and malignant oral disorders with increasing malignant potential Oral Microbiol Immunol 2009 24 4 314 8 10.1111/j.1399-302X.2009.00516.x 19572894

[B48] Postma TC Van Heerden WF Is the human papillomavirus a mutual aetiological agent in oral and cervical squamous cell carcinoma? Anticancer Res 2003 23 4 3509 12 12926099

[B49] Scully C Oral squamous cell carcinoma; from an hypothesis about a virus, to concern about possible sexual transmission Oral Oncol 2002 38 227 34 10.1016/S1368-8375(01)00098-7 11978544

[B50] Thomas M Pim D Banks L The role of the E6-p53 interaction in the molecular pathogenesis of HPV Oncogene 1999 18 7690 700 10.1038/sj.onc.1202953 10618709

[B51] Kadaja M Isok-Paas H Laos T Ustav E Ustav M Mechanism of genomic instability in cells infected with the high-risk human papillomaviruses PLoS Pathog 2009 5 4 10.1371/journal.ppat.1000397 19390600 PMC2666264

[B52] Holzinger D Halec G Schmitt M Pawlita M Bosh FX Molecular characterization of HPV16-associated squamous cell carcinomas of the oropharynx and larynx Oral Onc 2009 supplement 3 (1) 122 10.1016/j.oos.2009.06.284

[B53] Koskinen WJ Prevalence and physical status of human papillomavirus in squamous cell carcinomas of the head and neck Int J Cancer 2003 107 3 401 6 10.1002/ijc.11381 14506740

[B54] Mellin H Human papillomavirus type 16 is episomal and a high viral load may be correlated to better prognosis in tonsillar cancer Int J Cancer 2002 102 2 152 8 10.1002/ijc.10669 12385011

[B55] Peixoto T Castro PG Bussoloti Filho I Prevalence of human papillomavirus (HPV) in oral cavity and in oropharynx Rev Bras Otorinolaringol 2006 72 2 272 282 10.1016/S1808-8694(15)30068-9 PMC9445676 16951865

[B56] Remmerbach TW PCR detection of human papillomavirus of the mucosa: comparison between MY09/11 and GP5+/6+ primer sets J Clin Virol 2004 30 4 302 8 10.1016/j.jcv.2003.12.011 15163418

[B57] Morshed K Polz-Dacewicz M Szymajski M Dorota Polz Short-fragment PCR assay for highly sensitive broad-spectrum detection of human papillomaviruses in laryngeal squamous cell carcinoma and normal mucosa: clinico-pathological evaluation Eur Arch Otorhinolaryngol 2008 265 Suppl 1 S89 S96 10.1007/s00405-007-0569-5 18193443 PMC2441493

[B58] Eklund C Zhou T Dillner J The WHOHPV LabNet international proficiency study of HPV typing methods The 25th international Papillomavirus Conference: 8-14th May 2009, Malmö, Sweden This conforms to the requirements of the World Health Organization for the proficient detection of HPV DNA

[B59] Serrano M Hannon GJ Beach D A new regulatory motif in cell-cycle control causing specific inhibition of cyclin D/CDK4 Nature 1993 366 704 7 10.1038/366704a0 8259215

[B60] Sherr CJ Roberts JM CDK inhibitors: positive and negative regulators of G1-phase progression Genes Dev 1999 13 1501 12 10.1101/gad.13.12.1501 10385618

[B61] Jayasurya R Sathyan KM Lakshminarayanan K Phenotypic alterations in Rb pathway have more prognostic influence than p53 pathway proteins in oral carcinoma Mod Pathol 2005 18 1056 66 10.1038/modpathol.3800387 15731778

[B62] Fischer CA Kampmann M Zlobec I Green E Tornillo L Lugli A Wolfensberger M Terracciano LM p16 expression in oropharyngeal cancer: its impact on staging and prognosis compared with the conventional clinical staging parameters Ann Oncol 2010 21 10 1961 6 10.1093/annonc/mdq210 20423915

[B63] Reed AL Califano J Cairns P High frequency of p16 (CDKN2/MTS-1/INK4A) inactivation in head and neck squamous cell carcinoma Cancer Res 1996 56 3630 3 8705996

[B64] Burns JE Gene mutations and increased levels of p53 protein in human squamous cell carcinomas and their cell lines Br J Cancer 1993 67 6 1274 84 10.1038/bjc.1993.238 8390283 PMC1968513

[B65] Chang F Frequent mutations of p53 gene in oesophageal squamous cell carcinomas with and without human papillomavirus (HPV) involvement suggest the dominant role of environmental carcinogens in oesophageal carcinogenesis Br J Cancer 1994 70 2 346 51 10.1038/bjc.1994.305 8054284 PMC2033483

[B66] Goon PKC Stanley MA Ebmeyer J Steinsträsser L Upile T Jerjes W Bernal-Sprekelsen M Görner M Sudhoff HH HPV & head and neck cancer: a descriptive update Head & Neck Oncology 2009 1 36 10.1186/1758-3284-1-36 PMC2770444 19828033

[B67] Weinberger PM Molecular classification identifies a subset of human papillomavirus--associated oropharyngeal cancers with favorable prognosis J Clin Oncol 2006 24 5 736 47 10.1200/JCO.2004.00.3335 16401683

[B68] Gillespie MB Rubinchik S Hoel B Sutkowski N Human papillomavirus and oropharyngeal cancer: what you need to know in 2009 Curr Treat Options Oncol 2009 10 5-6 296 307 10.1007/s11864-009-0113-5 19768554

[B69] Jayasurya R Sathyan KM Lakshminarayanan K Phenotypic alterations in Rb pathway have more prognostic influence than p53 pathway proteins in oral carcinoma Mod Pathol 2005 18 1056 66 10.1038/modpathol.3800387 15731778

[B70] Pyeon D Newton MA Lambert PF den Boon JA Sengupta S Marsit CJ Woodworth CD Connor JP Haugen TH Smith EM Kelsey KT Turek LP Ahlquist P Fundamental Differences in Cell Cycle Deregulation in Human Papillomavirus-Positive and Human Papillomavirus-Negative Head/Neck and Cervical Cancers Cancer Research 2007 67 10 4605 4619 10.1158/0008-5472.CAN-06-3619 17510386 PMC2858285

[B71] Slebos RJ Yi Y Ely K Gene expression differences associated with human papillomavirus status in head and neck squamous cell carcinoma Clin Cancer Res 2006 12 3 Pt 1 701 9 10.1158/1078-0432.CCR-05-2017 16467079

[B72] Longworth MS Wilson R Laimins LA HPV31 E7 facilitates replication by activating E2F2 transcription through its interaction with HDACs EMBO J 2005 24 1821 30 10.1038/sj.emboj.7600651 15861133 PMC1142589

[B73] Li W Thompson CH Cossart YE The expression of key cell cycle markers and presence of human papillomavirus in squamous cell carcinoma of the tonsil Head Neck 2004 26 1 9 10.1002/hed.10335 14724900

[B74] Hoffmann M Gorogh T Gottschlich S Human papillomaviruses in head and neck cancer: 8 year-survival-analysis of 73 patients Cancer Lett 2005 218 199 206 10.1016/j.canlet.2004.09.027 15670897

[B75] Soares Christiane Pienna Neto Carlos Benatti Gabrielli Fregonezi Paula Andrea Teresa Debora Barreto de Macedo Santos Raimunda Telma Filho Adhemar Longatto Sakamoto Maeda Marina Yoshiê Computer-assisted analysis of p53 and PCNA expression in oral lesions infected with human papillomavirus Anal Quant Cytol Histol 2003 25 1 19 24 12630078

[B76] Zhao J Pestell R Guan JL Transcriptional Activation of Cyclin D1 Promoter by FAK Contributes to Cell Cycle Progression MBOC 2001 12 12 4066 4077 10.1091/mbc.12.12.4066 PMC60776 11739801

[B77] Cheung Y Chiu PM Tsun KL Khoo US Leung BSY Ngan HYS Chromosome in situ hybridisation, Ki-67, and telomerase immunocytochemistry in liquid based cervical cytology A N J Clin Pathol 2004 57 7 721 727 10.1136/jcp.2003.013730 PMC1770363 15220365

[B78] Holzinger D Halec G Schmitt M Pawlita M Bosch FX Molecular characterization of HPV16-associated squamous cell carcinomas of the oropharynx and larynx Oral abstracts/Oral Oncology 2009 Supplement 3 (1) 122 10.1016/j.oos.2009.06.284

[B79] Chuang AY Presence of HPV DNA in convalescent salivary rinses is an adverse prognostic marker in head and neck squamous cell carcinoma Oral Oncol 2008 44 10 915 9 10.1016/j.oraloncology.2008.01.001 18329326 PMC3215237

[B80] Zhao M Feasibility of quantitative PCR-based saliva rinse screening of HPV for head and neck cancer Int J Cancer 2005 117 4 605 10 10.1002/ijc.21216 15929076

[B81] Li Yang St John Maie AR Zhou Xiaofeng Kim Yong Sinha Uttam Jordan Richard CK Eisele David Abemayor Elliot Elashoff David Park No-Hee Wong David T Salivary Transcriptome Diagnostics for Oral Cancer Detection Clinical Cancer Research 2004 10 8442 8450 10.1158/1078-0432.CCR-04-1167 15623624

[B82] Xie Hongwei Onsongo Getiria Popko Jonathan de Jong Ebbing P Jing Cao Carlis John V Griffin Robert J Rhodus Nelson L Griffin Timothy J Proteomics Analysis of Cells in Whole Saliva from Oral Cancer Patients via Value-added Three-dimensional Peptide Fractionation and Tandem Mass Spectrometry Molecular & Cellular Proteomics 2008 7 486 498 10.1074/mcp.M700146-MCP200 18045803

[B83] Miah MS Crosbie RA Mountain RE Mahendran S The aetiopathogenesis of HPV in malignant disease: evidence for a global immunization programme Oral abstracts/Oral Oncology 2009 Supplement 3 (1) 121 122 10.1016/j.oos.2009.06.282

